# Gene family matters: expanding the HGNC resource

**DOI:** 10.1186/1479-7364-6-4

**Published:** 2012-07-05

**Authors:** Louise C Daugherty, Ruth L Seal, Mathew W Wright, Elspeth A Bruford

**Affiliations:** 1 European Bioinformatics Institute (EMBL-EBI), Wellcome Trust Genome Campus, Hinxton, Cambridgeshire, CB10 1SD, UK

## Abstract

The HUGO Gene Nomenclature Committee (HGNC) assigns approved gene symbols to human loci. There are currently over 33,000 approved gene symbols, the majority of which represent protein-coding genes, but we also name other locus types such as non-coding RNAs, pseudogenes and phenotypic loci. Where relevant, the HGNC organise these genes into gene families and groups. The HGNC website http://www.genenames.org/ is an online repository of HGNC-approved gene nomenclature and associated resources for human genes, and includes links to genomic, proteomic and phenotypic information. In addition to this, we also have dedicated gene family web pages and are currently expanding and generating more of these pages using data curated by the HGNC and from information derived from external resources that focus on particular gene families. Here, we review our current online resources with a particular focus on our gene family data, using it to highlight our new Gene Symbol Report and gene family data downloads.

## The HGNC: background and relevance

The HUGO Gene Nomenclature Committee (HGNC) has been responsible for approving unique and informative gene names and symbols to every human gene for over 30 years. Approved gene names and symbols preferably describe the structure, function or homology of a gene and its products. The provision of approved nomenclature allows researchers to discuss genes unambiguously, and this is reflected by HGNC symbol usage in scientific papers describing human genes, hence aiding the dissemination and interpretation of the associated data by the scientific community.

The HGNC website [1,2] provides direct links to genomic, proteomic and phenotypic information that is held in the HGNC database and enables users to search and download current data associated to their gene(s) of interest. As of February 2012, there are over 33,000 approved human gene symbols (including protein-coding genes, pseudogenes, ncRNA genes and phenotypes), each with a publicly available Gene Symbol Report. It is important to note that although the main focus of HGNC concerns human genes, there are coordinated efforts with other nomenclature committees [3], in particular the Mouse Genomic Nomenclature Committee (MGNC) [4] and Rat Genome Database (RGD) [5], and any large new gene family reorganisation or assignment is usually coordinated among these three nomenclature groups. The HGNC also regularly works with specialist advisors and publish scientific papers concerning gene family nomenclature and gene grouping [6-9]. The adoption of HGNC-approved gene names/symbols by the many genome browsers and databases reduces any uncertainty when referring to genes; for example, Ensembl [10], Entrez Gene [11], GeneCards [12], OMIM [13], UCSC [14], UniProt [15] and Vega [16] all use HGNC names/symbols. The data supplied by HGNC have been applied to a range of studies, such as assisting tools to identify candidate genes for further study [17], quantitative assessments of gene annotation status [18] and projects involving ‘mashups’ of bioinformatics data to explore genes involved in a particular disease [19] to name just a few.

## Website updates: the revamped genenames.org website

**Figure 1:**
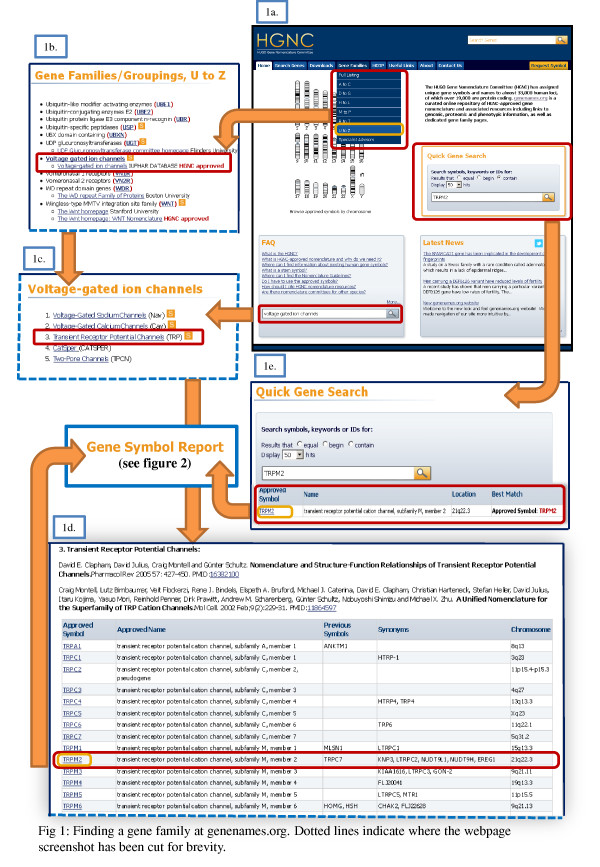
**Finding a gene family at genenames.org.** Dotted lines indicate where the webpage screenshot has been cut for brevity. (**1a**) Searching for a gene family on the HGNC homepage. (**1b**) Alphabetical listing of gene family names with links to the associated gene family pages. (**1c**) Large gene families are further divided into subgroups. (**1d**) Gene family pages and associated information are organised and represented by a table. (1e) Quick Gene Search tool is a quick way to find whether gene of interest is associated to a gene family.

Recently, we undertook a revamp of our website [1] to improve the navigation and access to our tools and data. The new HGNC homepage (Figure 1a) now has informative tabs with drop-down menus, enabling users to navigate to specific HGNC pages. The banner includes a simplified ‘Quick Gene Search’ that searches for terms that contain any part of the term and is present at the top of all pages. The ‘Quick Gene Search’ is also found on the homepage, and this allows users to search for terms that equal, begin or contain the search term and to select the number of returned results displayed [2]. At the bottom left of the homepage, there is now a HGNC website search box that allows searching of gene family pages and HGNC documentation and information pages. The homepage now has an interactive graphic of human chromosomes and mitochondrion, which allows users to ‘browse approved symbols by chromosome’. Each chromosome in the display is linked to the HGNC ‘Statistics and Downloads’ page, and the data returned are associated to the chromosome selected. This feature enables users to download the total number of approved symbols for the chromosome or browse symbols by locus type. Another recent addition to the homepage is the ‘News’ section that highlights any significant updates to the HGNC project and links to recent media articles that use approved gene symbols in their reports.

**Figure 2:**
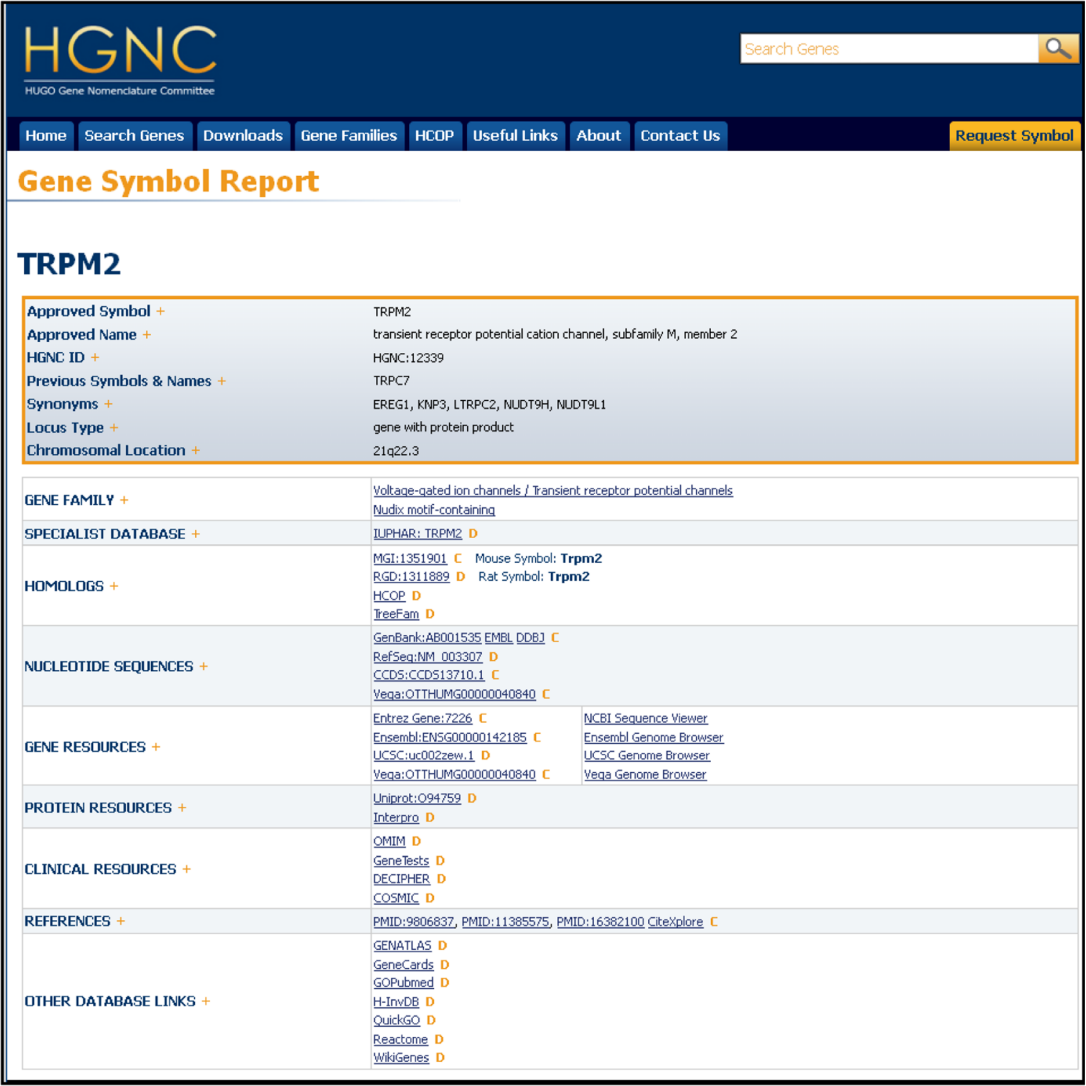
**Gene Symbol Report for*TRPM2.***

One of the main updates is the redesign of the Gene Symbol Report, which contains HGNC-curated data and data derived from external resources. All sections and corresponding links are described in full in our Symbol Report Documentation [20]. In this report, we will use *TRPM2* as an example to highlight the new features found in the report (Figure 2) and gene family pages (Figure 1).

We have restructured the Gene Symbol Report, so that the HGNC ‘core’ data are now more prominent and are presented in a separate table at the top of each report. The Gene Symbol Report for *TRPM2* (Figure 2) displays data in all the core fields. One of the most significant changes to our Gene Symbol Report is that we now provide access to the gene family page(s) linking from the ‘Gene Family’ name on the report to the associated gene family page. As shown in Figure 2*TRPM2* is associated to two gene families: the subgroup ‘Transient receptor potential channels’ and the ‘Nudix motif-containing family’. Gene families will be discussed in more detail later. Gene Symbol Reports also contain links to external biomedical resources. We have grouped related resources into the following sections: ‘Specialist Database’, ‘Homologs’, ‘Nucleotide Sequences’, ‘Gene Resources’, ‘Protein Resources’, ‘Clinical Resources’, ‘References’ and ‘Other Database Links’. External links can either be manually ‘curated’ by an HGNC curator, which is denoted by the letter ‘C’, or ‘downloaded’ from external sources, which is denoted by the letter ‘D’. The ‘Specialist Database’ section provides links to databases that are relevant to only certain classes of gene, and we now link to 14 specialist databases. In the example Gene Symbol Report for *TRPM2* (Figure 2), the specialist receptor database we link to is IUPHAR [21]. In the ‘Homologs’ section, in addition to linking to the mouse MGI [4] and rat RGD [5] databases, we now display the symbols approved by the two nomenclature committees. The nomenclature committees for human, mouse and rat aim to approve equivalent gene symbols and names for orthologous genes; for example, the human *TRPM2* Gene Symbol Report (Figure 2) shows that the approved symbols for mouse and rat are both *Trpm2*. Links to nucleotide sequences are grouped in the ‘Nucleotide Sequences’ section and include recently added links to Vertebrate Genome Annotation [16] gene sequence curated by the Havana project. The ‘Gene Resources’ section groups together links to the gene annotation pages at Entrez Gene [11], Ensembl [10], UCSC [14] and Vega [16]. As part of the Symbol Report redesign, we now also provide direct links to the Genome Browsers supported by these four projects. The ‘Protein Resources’ section still includes links to the UniProt project, but as part of the update, we have added a link to the InterPro [22] Protein Match page; this shows all predicted protein signatures (integrated and unintegrated) for the encoded protein by the InterPro member databases. All the mutation and variation-related data links are displayed in the ‘Clinical Resources’ section, while our curated links to references in PubMed [23] and CiteXplore [24] are shown in the ‘References’ section. Finally, the ‘Other Database Links’ section includes links to relevant biomedical resources that cannot be grouped into the categories above. For example, we now also link to the Reactome signalling pathway database [25] and to a list of all Gene Ontology terms annotated for the gene product at the QuickGO project [26].

## Gene families and groupings

Gene families are generally defined as a group of genes descended by duplication from a common ancestor. The degree of divergence from the ancestral gene can vary considerably between members, and they may or may not have a conserved function. In many cases, the homology between the family members may be restricted to a specific highly conserved region or domain(s) of the encoded protein, and genes can therefore belong to more than one gene family, e.g. *TRPM2* belongs to two gene families: the ‘Transient receptor potential channels’ and the ‘Nudix motif-containing family’ (see Figure 2). Large gene families may also be subdivided into smaller subfamilies, which often equate to functional groups. The ‘Transient receptor potential channels’ are a good example of this as they are subdivided into subfamily A, subfamily C, subfamily M and subfamily V [27].

When naming gene family members, the HGNC aims to use a common root (or stem) symbol which allows easy identification of the members, e.g. TRP is the root symbol for Transient receptor potential channels. As well as homologous gene families, the HGNC also organises human genes into gene groupings, which correspond to sets of genes that are not necessarily related by sequence homology but do have another shared feature: for example, a common function (e.g. ‘class I’ and ‘class II aminoacyl tRNA synthetases’), a specific chromosomal location (e.g. genes from the ‘pseudoautosomal region’), secondary structure (e.g. ‘micro RNAs’) or a grouping that provides a useful community resource (e.g. all genes encoding ‘blood group’ antigens). These groupings do not usually share a common root symbol, and again, one gene can be a member of more than one gene family and/or grouping. When assigning genes as members of families and groupings, the HGNC look at all available data including sequence similarity and conserved domain structure, publications and other databases, and where possible, take advice from specialist advisors who are experts working on that specific family or group. For ease of discussion, all gene families and groupings are referred to simply as gene families throughout this paper.

As of June 2012, close to 45% of the 33,000 HGNC database entries are associated with a gene family. The HGNC website [1] also currently displays over 237 curated webpages dedicated to individual gene families. However, if all the gene subfamilies are considered, this would give a larger total of around 400 pages due to instances where large gene families have been subdivided into smaller subgroups. The ‘Voltage-gated ion channels’ family (Figure 1c) is an example of a large gene family which has the following 11 subgroupings that make up the gene family: ‘Voltage-Gated Sodium Channels’, ‘Voltage-Gated Calcium Channels’, ‘Voltage-Gated Sodium Channels’, ‘CatSper’, ‘Two-Pore Channels’, ‘Cyclic Nucleotide-Regulated Channels’, ‘Calcium-Activated Potassium Channels’, ‘Voltage-Gated Potassium Channels’, ‘Inwardly Rectifying Potassium Channels’, ‘Two-P Potassium Channels’ and ‘Hydrogen Voltage-Gated Ion Channels’).

## Gene family resources: finding the information

HGNC is now in the process of actively expanding our gene family pages using internally curated data and data from the growing number of external resources and publications that focus on particular gene families. The HGNC homepage [1] (Figure 1a) features a website search box at the bottom of the page that allows searching for gene family pages, HGNC documentation and information pages. Furthermore, there are two main ways of ascertaining whether a gene is associated to a particular gene family: by browsing the ‘Gene Families’ pages [28] from the homepage or by directly querying the database by using one of the ‘Search Genes’ tools [29].

The ‘Gene Families’ [28] section from the drop-down menu on the homepage (Figure 1a.) gives an alphabetical listing of all the gene family names (Figure 1b) and links to the associated gene family pages. The information grouped on the gene family page is organised and represented by a table (Figure 1d), which lists all of the associated genes with the following data: ‘Approved Symbol’, ‘Approved Name’, ‘Previous Symbols’, ‘Synonyms’ and ‘Chromosome’. The ‘Approved Symbol’ links to the ‘Gene Symbol Report’, so selecting ‘TRPM2’ from the table will take the user to the specific Gene Symbol Report (Figure 2). The gene family page (Figure 1c,d) also indicates if there is a specialist advisor associated to the gene family, and their contact details, where applicable, are linked from each relevant gene family page by an orange ‘S’ icon. There are currently 115 specialist advisors that help with the content and approval of any new gene family members, and a separate page on the website lists all of HGNC's specialist advisors [30].

Users can also query the HGNC data by using the tools listed on the ‘Search Genes’ page [29]: the ‘Quick Gene Search’, ‘Advanced Gene Search’ and the ‘List Search’. These tools can all be used to obtain the Gene Symbol Report, so if the gene of interest has a gene family associated, it will link to the relevant gene family page from the ‘Gene Family’ name in the Gene Symbol Report. The ‘Quick Gene Search’ tool [31] (see Figure 1e) is a quick and easy way to check whether a gene of interest is associated to a gene family; this is done by querying the gene symbol, name or ID to locate a Gene Symbol Report. Using the ‘Advanced Gene Search’ [32] will allow the user to build a more specific query by choosing to query within specific datasets, e.g. only for those genes that are approved. The ‘List Search’ [33] also allows users to access the Gene Symbol Report but enables users to search multiple genes by gene symbol.

## Gene family resources: downloading the information

We have recently developed new and additional ways to download our gene family data, depending on the scope of data required. From the ‘Downloads’ tab, users can access the following:

· The ‘Statistics and Downloads’ page [34], which has a direct link to the ‘complete HGNC dataset’. This file includes all the HGNC core fields and now includes the new gene family data fields, ‘Gene Family Tag’ and ‘Gene Family Description’ (discussed below). Alternatively, if it is just the information relating to the gene family data that is required, select ‘complete HGNC Gene Family dataset’. This gives a file with the following fields: URL for gene family page, Gene Family Tag, Gene Family Description, Symbol and HGNC ID.

· The ‘Custom Downloads’ page, on the other hand, allows the specification of the exact fields required. The selection should include the ‘Gene Family Description’ and the ‘Gene Family Tag’ fields to retrieve the gene family data in the output.

· Finally, to obtain the data for the gene family associated to a particular gene of interest, select ‘Download gene family data’ beneath the gene family table on the relevant gene family page.

The data from our previously established ‘complete HGNC dataset’ and the newly created ‘complete HGNC Gene Family dataset’ can be associated together by the ‘Gene Family Tag’ field and the ‘Gene Family Description’ field. The ‘Gene Family Tag’ is used to generate gene family or group specific pages at the HGNC website [1] and does not necessarily reflect an official nomenclature. The ‘Gene Family Description’ is the name given to a particular gene family. Each ‘Gene Family Description’ has an associated ‘Gene Family Tag’ and vice versa. If a particular gene is a member of more than one gene family, the tags and the descriptions will be shown in the same order. Like all HGNC data, the ‘Gene Family’ datasets are updated daily, so the user will always get the most recent and up-to-date data when clicking on the download links or accessing custom downloads.

## Future directions

In the future, we plan to increase the number of gene families and the assignment of human genes to gene families. We will also arrange the current alphabetical gene family list into more meaningful categories, for example, grouping them by domain structure, function or disease associations. Another area we are considering is integrating the graphical display in InterPro [22] to represent the domains that are encoded by each gene family member.

Updates regarding the gene family pages are now also mentioned in our Twitter feed and quarterly ‘Newsletter’. If you subscribe to this, you will be notified of any new gene families; see our feedback form [35] and tick the box to receive the Newsletter. Alternatively, newsletters are also available on the website. If you have a gene family you think should be represented or you would like to be considered as one our specialist advisors, please contact us via hgnc@genenames.org.

## Competing interests

The authors declare that they have no competing interests.

## Authors’ contributions

LCD drafted the manuscript and created the new gene family pages. MWW, RLS and EAB curated the data in the HGNC dataset. All authors read and approved the final manuscript.
